# Facilitating education in pulmonary rehabilitation using the Living Well with COPD programme for pulmonary rehabilitation: a process evaluation

**DOI:** 10.1186/1471-2466-13-50

**Published:** 2013-08-05

**Authors:** Denise Cosgrove, Joseph MacMahon, Jean Bourbeau, Judy M Bradley, Brenda O’Neill

**Affiliations:** 1 Centre of Health and Rehabilitation Technologies (CHaRT), Institute of Nursing and Health research, School of Health Sciences, University of Ulster, Newtownabbey, BT37 OQB, Northern Ireland; 2 Department of Respiratory Medicine, Belfast City Hospital, Belfast, BT9 7AB, Northern Ireland; 3 Respiratory Epidemiology and Clinical Research Unit, Montreal Chest Institute, McGill University Health Centre, Montreal, H2X 2P4, Canada; 4 (Current affiliation) Northern Ireland Clinical Research Network (Respiratory Health), Belfast City Hospital, Belfast, BT9 7AB, Northern Ireland

**Keywords:** Pulmonary rehabilitation, COPD, Education, Self-management

## Abstract

**Background:**

Standardised evidenced-based materials and mechanisms to facilitate the delivery of the education component of pulmonary rehabilitation are not widely available. The aims of this study were: 1) to adapt the self-management programme Living Well with COPD (LWWCOPD) programme, for embedding in pulmonary rehabilitation; and, 2) to conduct a process evaluation of the adapted programme.

**Methods:**

The adaptations to the LWWCOPD programme were informed by focus groups, current practice, relevant research and guideline documents. Pulmonary rehabilitation sites used the adapted programme, the LWWCOPD programme for pulmonary rehabilitation, to deliver the education component of pulmonary rehabilitation. A process evaluation was conducted: elements included reach (patients’ attendance rates), dose delivered (amount of programme delivered), dose received (health professional and patient satisfaction) and fidelity (impact on patients’ knowledge, understanding and self-efficacy on the Understanding COPD questionnaire). Descriptive statistics (mean, SD) were used to summarise demographics and key data from the feedback questionnaires. Qualitative feedback on the programme was collated and categorised. Changes in the Understanding COPD questionnaire were examined using paired t-tests.

**Results:**

The LWWCOPD programme for pulmonary rehabilitation was delivered in eleven hospital- and community-based programmes (n=25 health professionals, n=57 patients with COPD). It consisted of six weekly 30–45 minute sessions. The process evaluation showed positive results: 62.3% of patients attended ≥ 4 education sessions (reach); mean (SD) 90 (10)% of the session content were delivered (dose delivered); the majority of sessions were rated as excellent or good by health professionals and patients. Patients’ satisfaction was high: mean (SD) Section B of the Understanding COPD questionnaire: 91.67 (9.55)% (dose received). Knowledge, understanding and self-efficacy improved significantly: mean change (95% CI): Section A of the Understanding COPD questionnaire: 26.75 (21.74 to 31.76)%, BCKQ 10.64 (6.92 to 14.37)% (fidelity).

**Conclusion:**

This rigorous process evaluation has demonstrated that the LWWCOPD programme for pulmonary rehabilitation can be used to deliver high quality, consistent and equitable education sessions during hospital and community-based pulmonary rehabilitation. This programme is now available worldwide (http://www.livingwellwithcopd.com/living-well-and-pulmonary-rehabilitation.html).

**Trial registration:**

This study was registered with clinicaltrials.gov (reference number: NCT01226836)

## Background

Pulmonary rehabilitation provides clinicians with an opportunity to deliver education and self-management skills to patients with chronic obstructive pulmonary disease (COPD) [1-4]. Recent surveys of pulmonary rehabilitation have reported variations in the content and delivery of education sessions between programmes [5-7]. Some groups have explored other self-management programmes within pulmonary rehabilitation, however, materials and mechanisms to deliver the important key information and self-management strategies specific to COPD are not widely available [8].

The “Living Well with COPD: A Plan of Action for Life” is an evidence-based self-management programme which is used extensively throughout Canada [9-11]. It consists of 1-1½ hours per week of education for 7 to 8 weeks either on a one-to-one basis or in a group setting [9]. An extensive range of teaching materials are available for health professionals and patients, and sessions range from basic information about COPD to integration of healthy behaviours and self-management strategies [12]. It was delivered as part of a chronic care programme in a multi-centre study. The LWWCOPD programme was delivered weekly on an individual basis over a 2-month period in patients’ homes, patients received weekly telephone calls for eight weeks and then monthly calls for the remainder of the 12-month study, case managers were also available by telephone for the duration of the study. The chronic care programme (including the LWWCOPD) significantly reduced hospital admissions, emergency room visits and unscheduled general practitioner visits and improved quality of life compared to usual care [9,10]. This chronic care programme (incorporating the LWWCOPD programme) has also been shown to result in statistically significant cost savings compared to usual care over a 12 month period [11]. Over time the LWWCOPD programme has been used in various settings but to date it has not been adapted to be delivered in the context of pulmonary rehabilitation. The LWWCOPD programme offers an opportunity to embed a well-structured education programme in the delivery of pulmonary rehabilitation. However, before it can be used in the context of pulmonary rehabilitation it requires adaptation.

Process evaluations are crucial in the development of complex interventions, such as education programmes [13]. They involve assessing the feasibility of delivering the intervention and its acceptance by providers and patients by identifying aspects of the programme that work well and aspects that require improvement [13]. The process evaluation model proposed by Saunders includes elements of reach (proportion of the intended audience that participates in the intervention, and their attendance rates), dose delivered (amount of the intervention delivered by facilitators), dose received (extent to which participants actively engage with, interact with and/or use materials/resources), and fidelity (extent to which the intervention was implemented as planned) [13-15]. The philosophy of the adapted programme is to provide health professionals with a mechanism to deliver key information and practical self-management strategies relevant to patients with COPD; and to enable patients to improve their knowledge and self-efficacy to help them manage their condition.

The aims of this study were to a) adapt the LWWCOPD programme for use in pulmonary rehabilitation and b) conduct a process evaluation of the adapted programme.

## Methods

### Adaptation of the LWWCOPD programme for pulmonary rehabilitation

The adaptation of the LWWCOPD programme for use in pulmonary rehabilitation followed a rigorous process over a 15 month period (January 2009 to March 2010). All modifications to the LWWCOPD programme were informed by focus groups, current practice, relevant research and guideline documents [1-4,7,16-19]. The focus groups of patients with COPD (n=32; six focus groups) and health professionals (n=8; one focus group) identified the key topics that they perceived were important for inclusion in the education component of pulmonary rehabilitation and how they preferred these to be delivered [16]. The key topics included disease education, management of breathlessness, management of an exacerbation, medications, psychosocial issues and welfare and benefits (Table 1). They preferred practical group-based education sessions delivered using visual aids and models and supplemented by written information. The content and materials of the LWWCOPD were compared to the findings of the focus groups. Gaps and differences in the programme warranted the development of new materials/content and the adaptation of existing materials/content. Experts and health professionals in their respective fields advised, collaborated on and reviewed individual sessions and/or specific information. All modifications and reformatting were reviewed in collaboration with the authors of the LWWCOPD programme who approved all final materials, verified the accuracy of and the evidence for the changes and ensured that they were within the spirit of the original LWWCOPD programme. Each of the modified education sessions was delivered to a lay population to establish length of time, ease of use and comprehension.

**Table 1:** Education sessions of the LWWCOPD programme for pulmonary rehabilitation

**Session number**	**Session title**
Session 1	Management of Breathlessness
Session 2	Energy Conservation
Session 3	Overview of the Action Plan and Management of an Exacerbation
Session 4	COPD Medication and appropriate use of Inhalation Devices
Session 5	Management of Stress, Anxiety and Depression
Session 6	Continuing Exercise and Self-Management Strategies

#### The Living Well with COPD programme for pulmonary rehabilitation

The LWWCOPD programme for pulmonary rehabilitation provides patients with disease-specific information and teaches self-management skills through the practical application of activities. The adapted programme consists of six weekly 30–45 minute sessions (Table 1). It is delivered using a range of educational materials and resources for both health professionals (introductory guide, health professional manuals [n=6], posters [n=25] and cue cards [n=6]), and patients (information booklet, key messages [n=5] and written action plan) (Additional file 1: Table S1).

The LWWCOPD programme for pulmonary rehabilitation was used by the health professionals to deliver the education component of a six-week pulmonary rehabilitation programme to the patients. Each pulmonary rehabilitation site delivered their usual exercise classes (with the exception of one programme), were delivered twice a week. The group-based education sessions were delivered after the exercise classes. All patients received the patient information booklet at the start of the pulmonary rehabilitation programme.

### Process evaluation of the LWWCOPD programme for pulmonary rehabilitation

#### Study design

The study included the assessment of elements of the process evaluation model proposed by Saunders: reach, dose delivered, dose received, and fidelity (Additional file 2: Table S2) [14]. Ethical approval was granted by the Office for Research Ethics Committees Northern Ireland (Reference: 09/NIR03/76) and governance approval was obtained from the relevant Health and Social Care Trusts. Data collection commenced in April 2010 and ended in October 2010.

#### Participants (health professionals and patients with COPD)

Health professionals who were involved in the delivery of education sessions in pulmonary rehabilitation based in hospital and community settings within three Health and Social Care Trusts in Northern Ireland were eligible to participate. Written consent was obtained from all health professionals. All health professionals attended a 3-hour training workshop to receive training in using the LWWCOPD for pulmonary rehabilitation materials, to practise delivering the education sessions and to receive information on the study methodology.

Patients were recruited from the pulmonary rehabilitation assessment clinics at each participating site. Patients who had a primary diagnosis of COPD as documented in medical and/or pulmonary rehabilitation notes, who had a good understanding of written English (as reported by the individual patient) and who were eligible for pulmonary rehabilitation based on a medical and clinical assessment were eligible to participate. Written consent was obtained from all patients. Patients who participated in the study attended pulmonary rehabilitation as part of a larger group (other attendees at pulmonary rehabilitation either declined the study or did not have COPD).

#### Data collection

##### Reach

The demographics of the patients were collected (gender, age, FEV_1_ percentage predicted, whether they received long-term oxygen therapy or previously attended pulmonary rehabilitation). Patients’ attendance rates were recorded each week. Adherence to the programme was considered to be attendance at four or more education sessions [20].

##### Dose delivered

Data were gathered from health professionals on the number of education sessions from the programme that were delivered, the duration of the education sessions, the percentage of the session contents that were covered and whether they displayed/disseminated the patient materials.

##### Dose received

After each education session the health professional who delivered the session and the patients who attended the session completed written feedback questionnaires [21]. Five-point Likert scales were used to assess the health professionals’ and patients’ overall opinion of the session (range: excellent to poor) and their satisfaction with the amount of practical information in the session (range: very satisfied to very unsatisfied). Health professionals rated their satisfaction with the materials provided to deliver the session (range: very satisfied to very unsatisfied). Health professionals’ opinions of the advantages and disadvantages of the programme and patients’ opinions of aspects of the session they most and least enjoyed were gathered. Both groups were asked to suggest areas for improvement.

After pulmonary rehabilitation patients completed Section B (6 questions) of the Understanding COPD (UCOPD) questionnaire which assesses their satisfaction with the education component of pulmonary rehabilitation [22,23].

##### Fidelity

Health professionals’ feedback on how they used the content and materials and their views on using programme to deliver key information and self-management strategies relevant to patients were gathered using written feedback questionnaires. Changes in patients’ knowledge and self-efficacy to manage their condition were measured using Section A of the UCOPD questionnaire and the Bristol COPD Knowledge questionnaire (BCKQ) pre- and post-pulmonary rehabilitation [22-24]. Patients views were also gathered using feedback questionnaires.

### The UCOPD questionnaire

The UCOPD questionnaire is an 18-item, self-administered, COPD-specific questionnaire which is designed to assess patients’ perception of their understanding of, self-efficacy with, and use of key self-management skills (Section A; Domains: About COPD, Managing Symptoms of COPD, Accessing Help and Support) [21]. It also has a short section (Section B; 6 questions) which assesses patients’ satisfaction with the education component of pulmonary rehabilitation. Responses to each question are recorded using a visual numeric scale, which is anchored with word descriptors and has intervals scored from 0 to 10. The patient is instructed to circle the number on the scale which represents their understanding, confidence, use or satisfaction of/with each topic [22,23]. The scores of the domains and sections are calculated by summating the scores of the individual questions of that domain/section (minimum score of all domains/sections = 0; maximum scores: About COPD domain = 70, Managing Symptoms of COPD domain = 70; Accessing Help and Support domain = 40; Section A = 180, Section B = 50). The scores are then converted to percentages. Higher scores represent greater understanding, confidence and use of self-management skills and satisfaction with the education component of pulmonary rehabilitation.

The UCOPD questionnaire has been shown to have good practical and psychometric properties [22,23]. The questionnaire and instructions for administration and collating the results are readily available.

### The BCKQ

The BCKQ assesses knowledge and has also been shown to have good psychometric properties [24]. It is a self-administered, multiple-choice questionnaire. It has 13 topics, each of which contains five statements. Patients respond to the statements with “true”, “false” or “don’t know”. A correct response receives a score of 1, while an incorrect or “don’t know” response receives a score of zero. The total score is calculated by summating the number of correct responses (minimum score = 0; maximum score = 65). The scores can then be converted to percentages. Higher scores represent greater knowledge.

#### Data analysis

Analysis was conducted using SPSS Version 17. Descriptive statistics (mean, standard deviation [SD]) were used to summarise demographics, attendance rates and key data from the feedback questionnaires. Comments and responses to open questions were collated and the research team grouped together those with similar themes.

Normal distribution of the data was explored using the Kolmogorv-Smirnov statistic. Chi-square and independent t-tests were used to examine differences between the demographics and baseline scores of adherent and non-adherent patients. Paired-samples t-tests were used to examine changes in the UCOPD questionnaire and the BCKQ following pulmonary rehabilitation. The eta squared statistic was calculated to establish effect sizes and was classified using Cohen’s recommendations: 0.01 represented a small effect size, 0.06 a moderate effect size and 0.14 a large effect size [25]. Independent-samples t-tests were used to compare the change scores (of Section A of the UCOPD questionnaire and the BCKQ) of adherent and non-adherent patients following pulmonary rehabilitation. The Mann–Whitney U Test was used to compare the scores of adherent and non-adherent patients for Section B (satisfaction) of the UCOPD questionnaire.

## Results

### Adaptation of the LWWCOPD programme for pulmonary rehabilitation

Adaptations made to the original LWWCOPD programme included reducing the number of education sessions from eight to six to reflect the core topics patients had identified as important for pulmonary rehabilitation [16] and reducing the length of the education sessions from 1-1½ hours to 30–45 minutes to reflect current practice of pulmonary rehabilitation in the United Kingdom [5,7]. Terminology was reviewed and localised to improve readability and applicability of information. Cue cards were developed to display during the exercise classes to help integrate key self-management skills (for example, pursed lip breathing and pacing) [26]. Key message summary sheets were developed for patients to summarise each education session to help increase information uptake and memory recall [26]. A written action plan for COPD was developed based on the original LWWCOPD programme [9,27].

### Process evaluation of the LWWCOPD programme for pulmonary rehabilitation

The LWWCOPD programme was delivered during eleven pulmonary rehabilitation programmes at eight different out-patient pulmonary rehabilitation sites in hospital and community settings (hospital-based n=4; health centre n=1; community centre n=1; leisure centre n=2). Each education session was delivered by a single health professional. A total of 25 health professionals were involved in the delivery of education in this study. A range of disciplines were involved: nurses, physiotherapists, occupational therapists, a doctor and a pharmacist. The mean number of health professionals who delivered education sessions in each site was 3 health professionals (SD: 1; range: 2–5). The disciplines of the health professionals who delivered the education varied between sites depending on the skill mix available.

#### Reach

**Figure 1:**
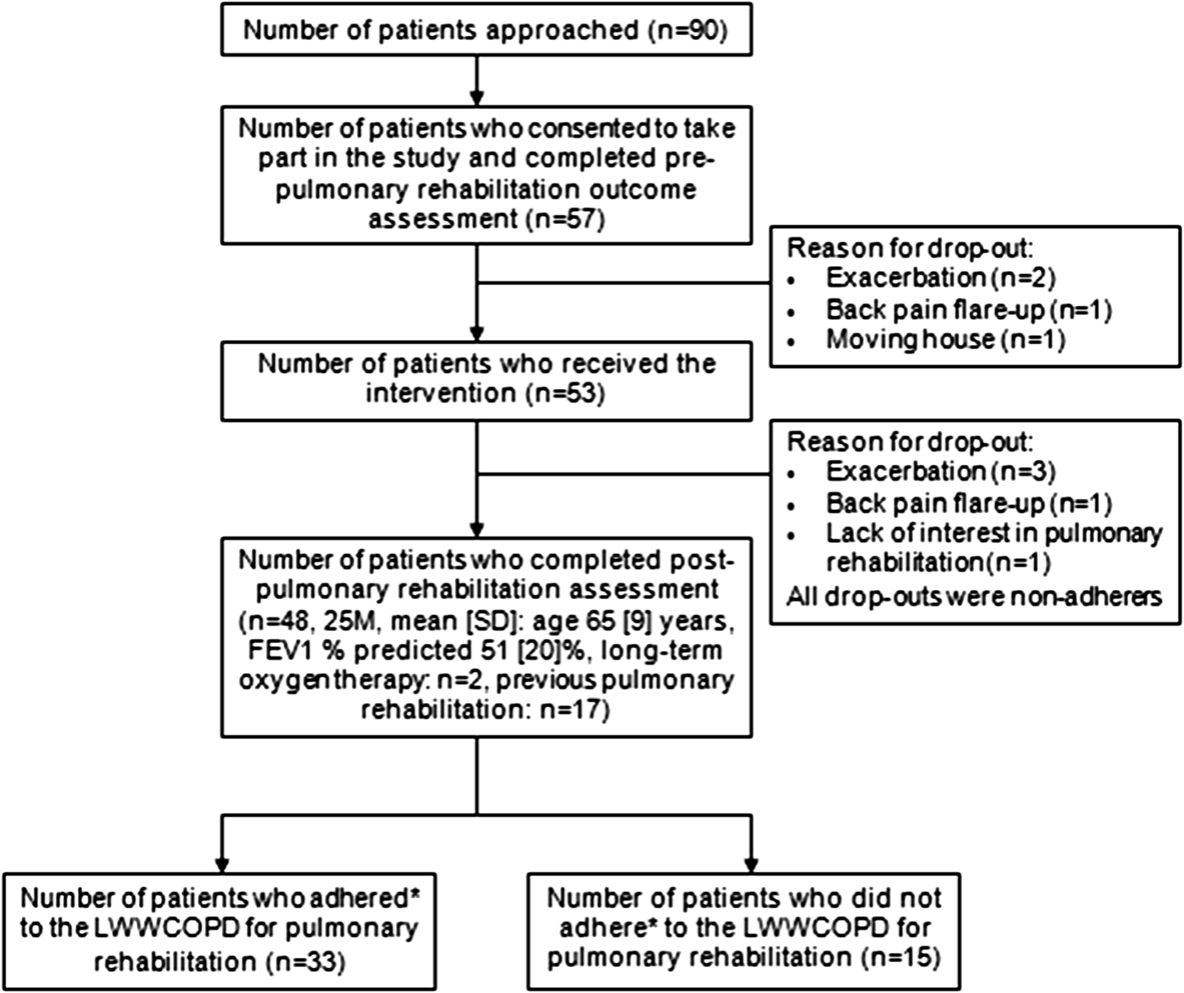
**Flow diagram showing the number of patients recruited to evaluate the LWWCOPD programme for pulmonary rehabilitation.** Key: Adherence was defined as attendance at four or more education sessions.

Ninety patients with COPD attending the pulmonary rehabilitation clinics met the study inclusion/exclusion criteria (Figure 1). Fifty seven patients consented to the study, of which 53 patients (27 males, mean [SD] age 65 [10] years, FEV_1_ predicted 50 [19] %, airflow obstruction: mild n=5, moderate n=21, severe n=19, very severe n=8, on long-term oxygen therapy: n=2, attended pulmonary rehabilitation previously: n=17) commenced the programme. They attended the programme as part of a larger group (other attendees either declined the study or did not have COPD). Post-pulmonary rehabilitation outcomes were completed by 48 patients (n=48/53; 91%). The demographics of the patients who attended the programmes were typical of the population for whom pulmonary rehabilitation is recommended for.

The mean number of patients attending each class was 6 patients (SD: 2; range 3–13). Thirty-three patients adhered to the programme (attended ≥ 4 education sessions) (n=33/53; 62.3%). The mean (SD) attendance of adherers and non-adherers were 5 (1) education sessions and 2 (1) education sessions respectively. Eleven patients attended all six sessions. There were no significant differences between adherent and non-adherent patients in key demographics including age, gender and severity of airflow obstruction. Reasons given for non-adherence included: co-morbidities, for example flare-up of arthritis, fibromyalgia, gout (n=4), clashed with other commitments, for example family commitments, work commitments, clinic appointments for co-morbidities, holidays (n=4), transport issues (n=2), lack of interest in pulmonary rehabilitation (n=2) and pulmonary exacerbation (n=1). Two patients did not give a reason.

#### Dose delivered

All six education sessions were delivered during each pulmonary rehabilitation programme (total number of sessions: n=66, completed evaluation questionnaires obtained: n=65). The 65 education sessions lasted for a mean (SD) 41 (8) minutes. The amount of content from the health professional manual covered during the education sessions was: mean (SD) 90 (10)%. The cue cards were displayed during the exercise component prior to most of the education sessions (n=54/65, 83%), and the key messages/action plan were distributed to patients during 94% education sessions (n=61/65, 94%).

#### Dose received

The health professionals reported good acceptability of the programme; the majority of the 65 education sessions were rated as either excellent (n=16/65, 25%) or good (n=40/65, 62%) and they were satisfied with the amount of practical information included in the sessions (n=57/65, 88%). The health professionals were either very satisfied or satisfied with the written materials provided: health professional manual, 95%; posters, 91%; key messages, 89%; action plans, 90%; and cue cards, 77%. The health professionals provided positive feedback on the content of the sessions, design of the materials and the delivery of the programme (Table 2; Additional file 3: Table S3). They also suggested areas of the sessions/programme for improvement, for example the inclusion of an education session on Healthy Eating (Table 2; Additional file 3: Table S3).

**Table 2:** Health professionals’ feedback on the LWWCOPD programme for pulmonary rehabilitation

**Feedback**	
**Positive feedback**	**Negative feedback / Areas for improvement**
Good resources for the health professional	Repetitive
Comprehensive, evidence-based content	Long sessions
Good patient friendly materials	Poor materials
Variety of teaching methods	Too many medical terms
Structured, distinct education sessions with defined curriculum	Not suitable for other conditions
Can be delivered by any health professional	Very scripted
Easier to deliver for the second time	Requires preparation and practice
	Order of education sessions
	Group dynamics
	Content

The feedback of all 53 patients who attended 203 education sessions was included in the analysis. In general, the patients reported good acceptability: the majority of the education sessions were rated as either excellent (n=143/203, 70.4%) or good (n=56/203, 27.6%). Patients were satisfied with the amount of practical information in all education sessions (n=203/203). Feedback on aspects of the programme patients enjoyed and areas of the programme they suggested for improvement are detailed in Table 3 (also in Additional file 4: Table S4).

**Table 3:** Patients’ feedback during the evaluation of the LWWCOPD programme for pulmonary rehabilitation

**Component**	**Feedback**
Improved knowledge and self-efficacy	Managing COPD
	Managing breathlessness
	Conserving energy
	Managing exacerbations
	Taking medications
	Managing psychosocial issues
	Taking part in exercise
Peer support	Meeting other people with COPD
	Sharing of information
Staff /Atmosphere	Friendly, approachable and helpful
	Fun/enjoyable
Content	Clear, understandable and useful information
	Interactive and practical demonstrations
	Visual reinforcement
Suggestions for improvement	Delivery
	Content
	Location
	Length of sessions
	Facilitators
	Supplementary materials
	Inclusion of family
	Additional sessions

Satisfaction reported on Section B of the UCOPD questionnaire was generally high; the mean (SD) total score was 91.67 (9.55)%. The mean (SD) score of the individual questions were (out of a maximum score of 10): satisfaction with practical information 9.31 (0.99); satisfaction with content of education sessions 9.44 (0.94); satisfaction of content of written information 9.25 (1.19); approachability of the health professionals 9.75 (0.60); accessibility of location 8.08 (2.55). Only one patient suggested an additional topic that they thought should be covered during the education sessions (relaxation techniques).

The health professionals’ and patients’ suggested areas for improvement were used to evolve the programme and the health professional training workshop. Based on feedback received some minor improvements were made to the content of some sessions; for example, the addition of using short-acting bronchodilators during an acute attack of breathlessness. Further lay terms were incorporated into the materials. Additional posters and cue cards were developed, existing posters and cue cards were made more visual by adding illustrations and they were printed larger. Additional education sessions were developed at the request of both health professionals and patients. New topics include Long-Term Oxygen Therapy, Airway Clearance, Healthy Eating and Smoking Cessation. The training workshop now covers when it is appropriate to adlib during sessions, how to modify content for non-COPD patients, and the importance of preparing and practicing prior to use. The flexible nature of the programme is also covered, for example, if required an education topic can be delivered over two sessions. The importance of involving family members and optimising the accessibility of the venue is also emphasised.

#### Fidelity

Although we did not directly observe the delivery of the education sessions feedback from the health professionals indicated that they delivered the programme as trained in terms of content and use of materials.

Health professionals’ feedback indicated that the LWWWCOPD for pulmonary rehabilitation was useful and comprehensive for the delivery of key information and self-management skills to patients with COPD (Table 2; Additional file 3: Table S3).

There was a statistically significant improvement in each domain and the total score of Section A of the UCOPD questionnaire, with large effect sizes following pulmonary rehabilitation (Table 4). There was also a statistically significant improvement in the BCKQ, with a large effect size (Table 4). Feedback from patients also supported the improvement in knowledge and self efficacy (Table 3; Additional file 4: Table S4).

**Table 4:** Paired-samples t-tests comparing pre- and post-pulmonary rehabilitation scores of the UCOPD questionnaire and the BCKQ (n=48)

	**Mean change (95% CI)**	**Effect size**	**P value**
**About COPD domain (%)**	23.78	0.61	<0.001
	(18.16 to 29.40)		
**Managing Symptoms of COPD domain (%)**	26.64	0.68	<0.001
	(21.24 to 32.04)		
**Accessing Help and Support domain (%)**	32.14	0.57	<0.001
	(23.91 to 40.36)		
**Section A total score (%)**	26.75	0.71	<0.001
	(21.74 to 31.76)		
**BCKQ (%)**	10.64	0.41	<0.001
	(6.92 to 14.37)		

**Key:** Understanding COPD questionnaire: Section A and domains: Minimum score = 0%, maximum score = 100% (higher score = greater understanding, confidence and use of self-management skills). Bristol COPD Knowledge Questionnaire: Minimum score = 0%, maximum score = 100% (higher score = greater knowledge).

There were no significant differences between the change scores of adherent (n=33) and non-adherent (n=15) patients in each domain and the total score of Section A of the UCOPD questionnaire or the BCKQ. There was also no significant difference between adherers and non-adherers in satisfaction (for the individual items or total score of Section B, UCOPD questionnaire).

## Discussion

This paper describes the successful adaptation of the LWWCOPD programme for use in pulmonary rehabilitation, and summarises the findings of a comprehensive process evaluation of the adapted programme. Results indicated that the self-management programme had good reach: patients had good attendance at the programme. Dose delivery and dose received were good: the programme was feasible to deliver across a range of pulmonary rehabilitation settings (community and hospital) and was well accepted by health professionals and patients who identified areas of the programme that worked well. Although health professionals’ delivery of the programme was not directly observed it can be inferred that the fidelity of the sessions were adhered to as patients showed improved knowledge, understanding and self-efficacy after the programme. This process evaluation study also identified areas of the programme that could be improved from the perspectives of health professionals and patients.

Pulmonary rehabilitation should promote lasting health behaviour and lifestyle change [1]. Previous studies investigating education programmes in COPD have found varying degrees of effectiveness [9,17,28]. It is difficult to ascertain why differences in effectiveness exist between studies but incorporating process evaluations in future studies of educational programmes may help to identify the aspects of programmes which contribute to successful outcomes [13,29]. Although other education programmes exist for pulmonary rehabilitation none have undergone an extensive process evaluation [30].

There are notable strengths of the LWWCOPD programme for pulmonary rehabilitation. It was developed to meet service needs and incorporated research involving the typical patient population that pulmonary rehabilitation is recommended for as well as the typical health professionals who are routinely involved in the delivery of pulmonary rehabilitation programmes [1-7]. An iterative process was used when adapting and developing it [7,16,17]. Any areas for improvement which were identified through the process evaluation study have since been incorporated into the programme. For example, additional topics have now been developed such as Healthy Eating and Smoking Cessation; each of these has undergone the same rigorous development as the other topics. Health professionals complemented the flexible scripted nature of the LWWCOPD programme for pulmonary rehabilitation, and this enables individual or multiple members of the multidisciplinary respiratory team with adequate skills and training to deliver the key education sessions. It is versatile as it can be tailored to the individual’s and/or group’s needs by delivering additional educational topics, or by delivering any one of the topics across two shorter education sessions. All materials are available on the LWWCOPD website which will facilitate free international access and easy updates to the materials in response to future changes in clinical guidelines (http://www.livingwellwithcopd.com/living-well-and-pulmonary-rehabilitation.html; health professional password: COPD; patient password: livingwelluk) [12,26]. This will facilitate the integration of the programme into a diverse range of healthcare systems at minimum cost.

The observed improvements in patient knowledge, understanding and self-efficacy following the LWWCOPD programme for pulmonary rehabilitation reflect the results of previous pulmonary rehabilitation cohort studies [24,31-35]. The magnitude of change in the BCKQ following pulmonary rehabilitation in this study (effect size: 0.41) was similar to the magnitude of change calculated from other studies of pulmonary rehabilitation (effect size: 0.68, 0.26) [24,36] and disease-specific education (effect size: 0.48) [37]. High levels of patient satisfaction were noted and this is vital to the success of rehabilitation because active participation in rehabilitation is fundamental to physical improvement and learning new skills [38].

Interestingly there were no significant differences between adherent and non-adherent patients in the amount of observed improvement in knowledge and self-efficacy however this study was not powered to explore differences in adherers and non-adherers. A recent cohort study in pulmonary rehabilitation found that the knowledge levels of completers (patients who attended all six education sessions) improved significantly following pulmonary rehabilitation, whereas non-completers (patients who did not attend all sessions) did not change [34]. Our findings may be explained by the philosophy of LWWCOPD for pulmonary rehabilitation which embeds a number of strategies to facilitate carryover between education sessions and also between exercise and education. For example, irrespective of adherence all patients got the LWWCOPD for pulmonary rehabilitation patient information booklet and the key messages from each education session. Additionally the cue cards are displayed during the exercise classes to re-enforce key self-management skills from the education sessions.

The LWWCOPD programme for pulmonary rehabilitation was evaluated in 57 patients with COPD, typical of the population that should be offered pulmonary rehabilitation recommended by international guidelines and that have been included in other pulmonary rehabilitation studies [32]. Although the programme was evaluated in the UK and in a range of settings, we did not identify any factors or issues which could affect implementation in a wider variety of settings and/or health care systems. It was developed specifically for the COPD population, but many elements of the programme are relevant to patient groups who present with symptoms, experiences and challenges similar to patients with COPD [1]. Future research could adapt and assess the LWWCOPD programme for pulmonary rehabilitation in other respiratory populations, or compare outcomes of the LWWCOPD for pulmonary rehabilitation programme to the use of generic self-management programmes within pulmonary rehabilitation such as the Chronic Disease Self-Management Programme (CDSMP) [8].

## Conclusion

In conclusion, this rigorous process evaluation has demonstrated that the LWWCOPD for pulmonary rehabilitation can be used to deliver high quality, consistent and equitable education sessions during hospital and community-based COPD pulmonary rehabilitation.

## Abbreviations

BCKQ: Bristol COPD knowledge questionnaire; COPD: Chronic obstructive pulmonary disease; FEV1: Forced expiratory volume in one second; LWWCOPD: Living well with COPD; SD: Standard deviation; UCOPD: Understanding COPD questionnaire

## Competing interests

The authors declare that they have no competing interests.

## Authors’ contributions

DC contributed to the study design, development of the intervention, training of health professionals, data acquisition, data analysis and interpretation, revision of the intervention based on study results and preparation of the manuscript. JM contributed to the study design, development of the intervention, revision of the intervention based on study results and critical review and revision of the manuscript. JBourbeau contributed to the development of the intervention, revision of the intervention based on study results and critical review and revision of the manuscript. JBradley contributed to the study design, development of the intervention, training of health professionals, data acquisition, data analysis and interpretation, revision of the intervention based on study results and preparation of the manuscript. BON contributed to the study design, development of the intervention, training of health professionals, data acquisition, data analysis and interpretation, revision of the intervention based on study results and preparation of the manuscript. All authors approved the final manuscript.

## Authors’ information

The adaptation and evaluation of the LWWCOPD for embedding in pulmonary rehabilitation formed part of DC’s PhD programme of research. Through a one-year post-doctorate post DC facilitated the translation of the programme into clinical practice in Northern Ireland by training all pulmonary rehabilitation programmes (n=27) in the use of the programme and supplying each pulmonary rehabilitation site with a complete copy of the programme.

BO’N is a member of the Northern Ireland Regional Respiratory Forum which developed the Service Framework for Respiratory Health and Wellbeing. Patient access to pulmonary rehabilitation and self management education are two of the core standards of the Framework. Therefore, it is imperative that this programme is translated to facilitate clinicians to meet the Frameworks’ core standards.

DC, JBradley and BO’N are members of the Association of Chartered Society of Physiotherapists (ACPRC). The ACPRC is the largest grouping of Respiratory Physiotherapists in the United Kingdom. The LWWCOPD programme for Pulmonary Rehabilitation is endorsed by the ACPRC. Publication would assist in further disseminating this programme.

In 1996, JBourbeau initiated steps to develop, evaluate and implement a self-management program specific to COPD, the Living Well with COPD (LWWCOPD). This program was to ensure optimal management of COPD, continuity of care, development of a global care approach focusing on patients and their family, standardisation of care in specialised clinics as well as first line service. There are multiple publications evidencing the beneficial impact of the LWWCOPD programme. JBourbeau collaborated with the UK group to adapt the LWWCOPD programme especially for use in pulmonary rehabilitation.

Judy M Bradley and Brenda O’Neill are joint senior authors.

## Pre-publication history

The pre-publication history for this paper can be accessed here:

http://www.biomedcentral.com/1471-2466/13/50/prepub

## Supplementary Material

Additional file 1: Table S1 Materials of the Living Well with COPD programme for pulmonary rehabilitation.
Click here for file

Additional file 2: Table S2 Elements of the process evaluation model assessed.
Click here for file

Additional file 3: Table S3 Health professionals’ feedback on the LWWCOPD programme for pulmonary rehabilitation.
Click here for file

Additional file 4: Table S4 Patients’ feedback during the evaluation of the LWWCOPD programme for pulmonary rehabilitation.
Click here for file
