# Supplementary material for: Facilitating education in pulmonary rehabilitation using the Living Well with COPD programme for pulmonary rehabilitation: a process evaluation
Source: BMC Pulm Med. 2013 Aug 5;13:50. doi: 10.1186/1471-2466-13-50 (PMC3751129; doi:10.1186/1471-2466-13-50)
Supplement: Additional file 1: Table S1 — Materials of the Living Well with COPD programme for pulmonary rehabilitation. [file 1471-2466-13-50-S1.doc]

**Additional file 1: Table S1** Materials of the Living Well with COPD programme for pulmonary rehabilitation

| **Material** | **Description** |
| --- | --- |
| 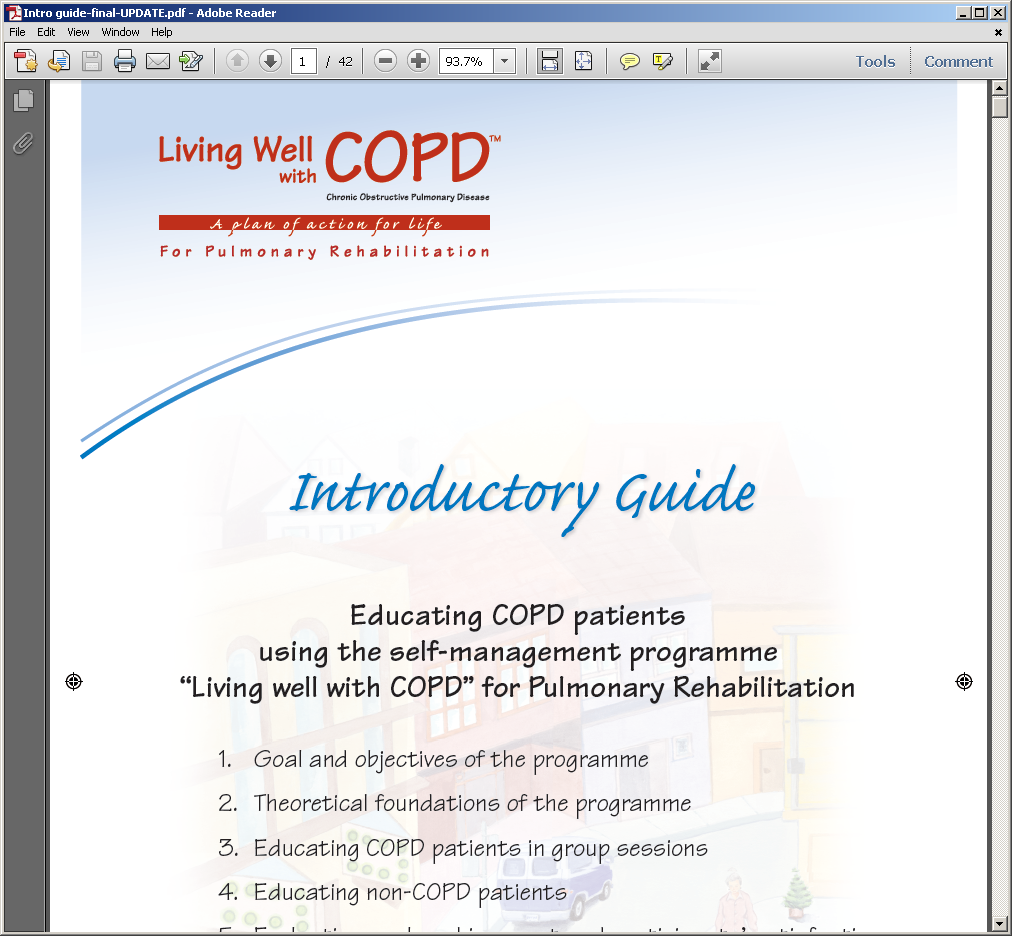 | **Health Professional Introductory Guide:** This is a “Background resource” that covers the goals and theory of the programme, and of delivering education to adults and dealing with group dynamics. |
| 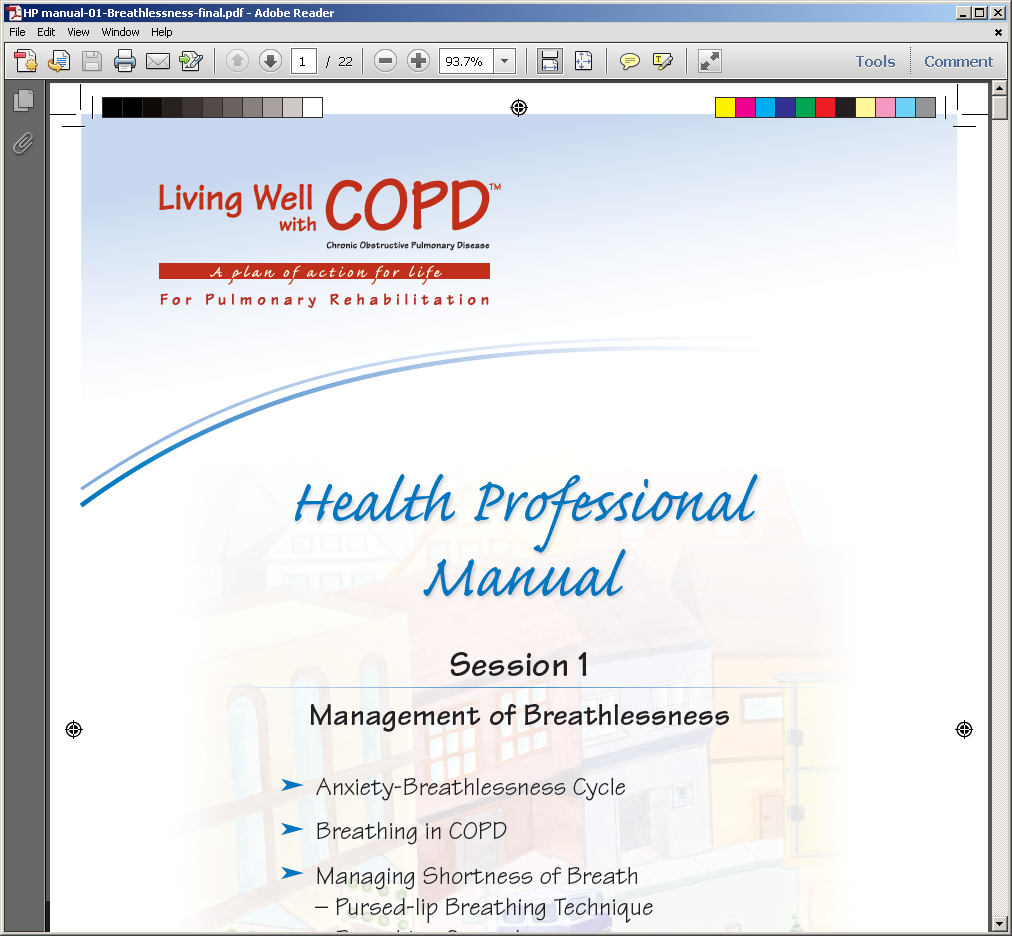 | **Health Professional manual:** These 6 manuals provide the health professionals with a script and step-by-step guide through the delivery of the education session. 1. Management of breathlessness, 2. Energy conservation, 3. Management of an exacerbation, 4. Medications and inhalers, 5. Stress, anxiety and depression, 6. Continuing exercise and self-management strategies *(including welfare and benefits).* |
| **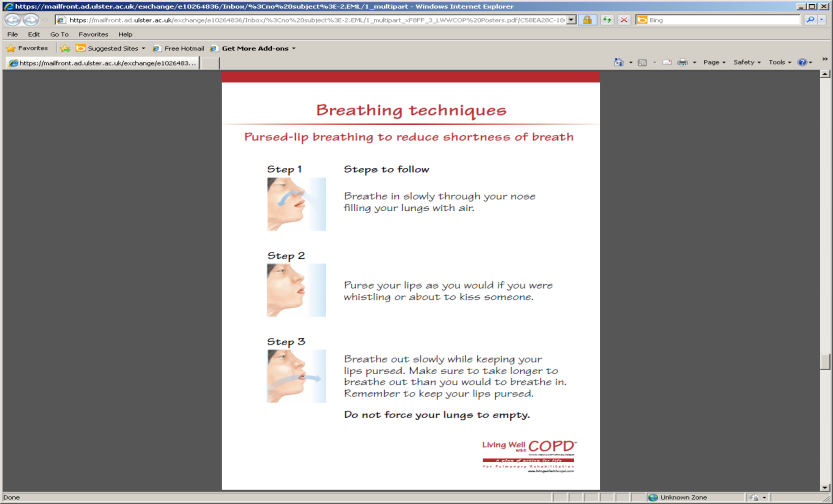** | **Posters:** There are 25 posters which act as visual cues and support the information delivered during each session. |
| 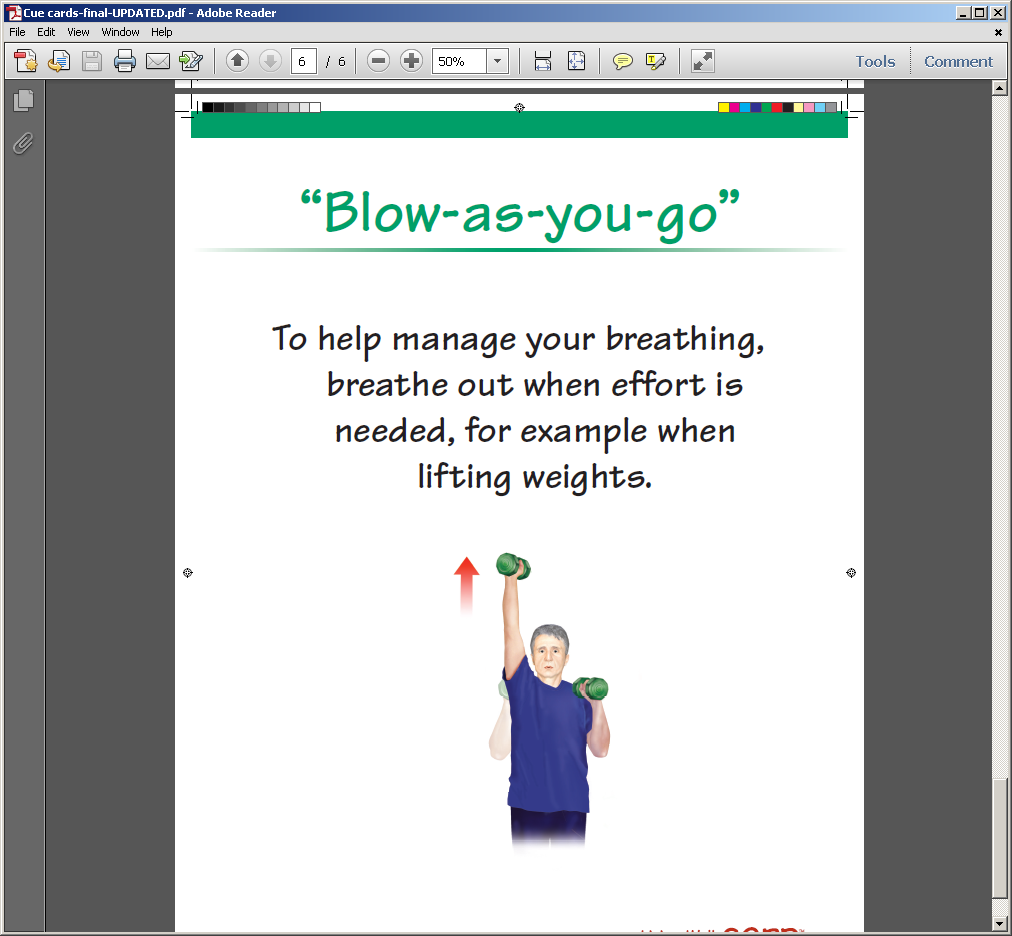 | **Cue cards:** There are six cue cards which are displayed during the exercise class. These translate some of the key messages covered during the education sessions into the exercise class. |
| 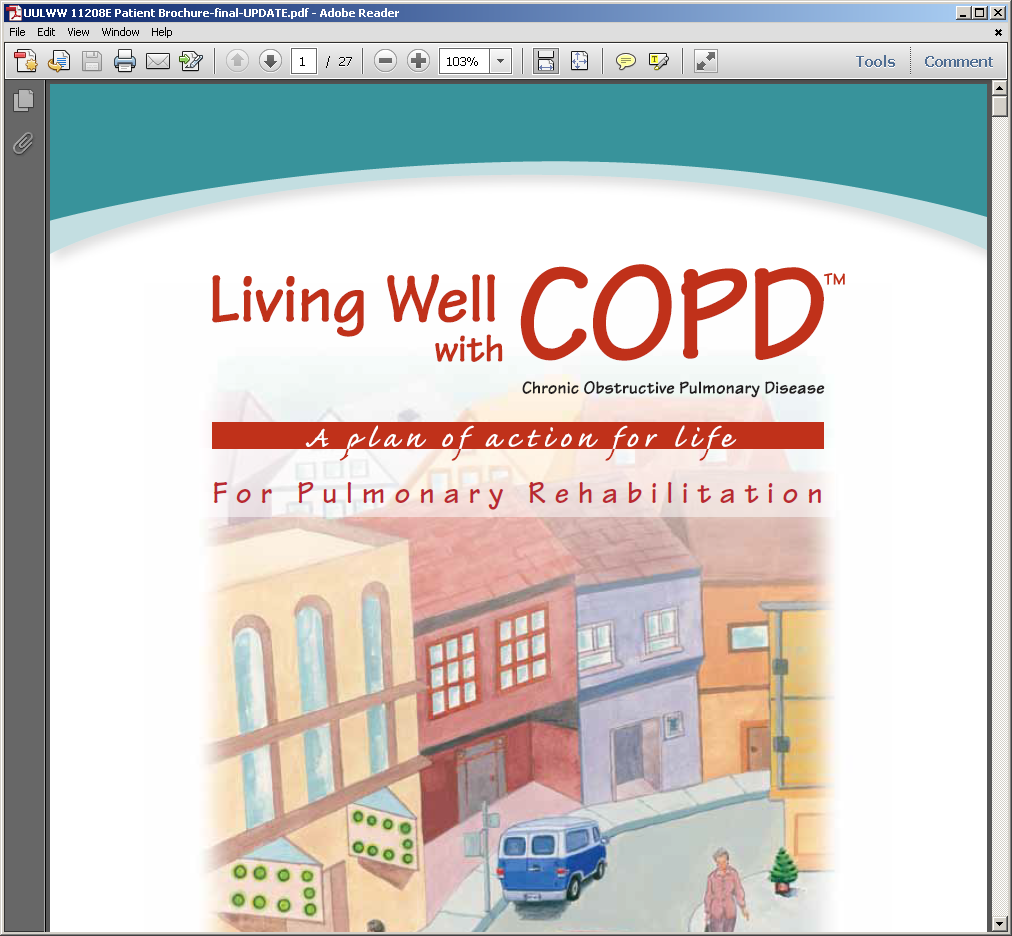 | **Patient booklet:** This patient booklet provides the information covered during the education sessions and is a resource for the patients to keep. |
| 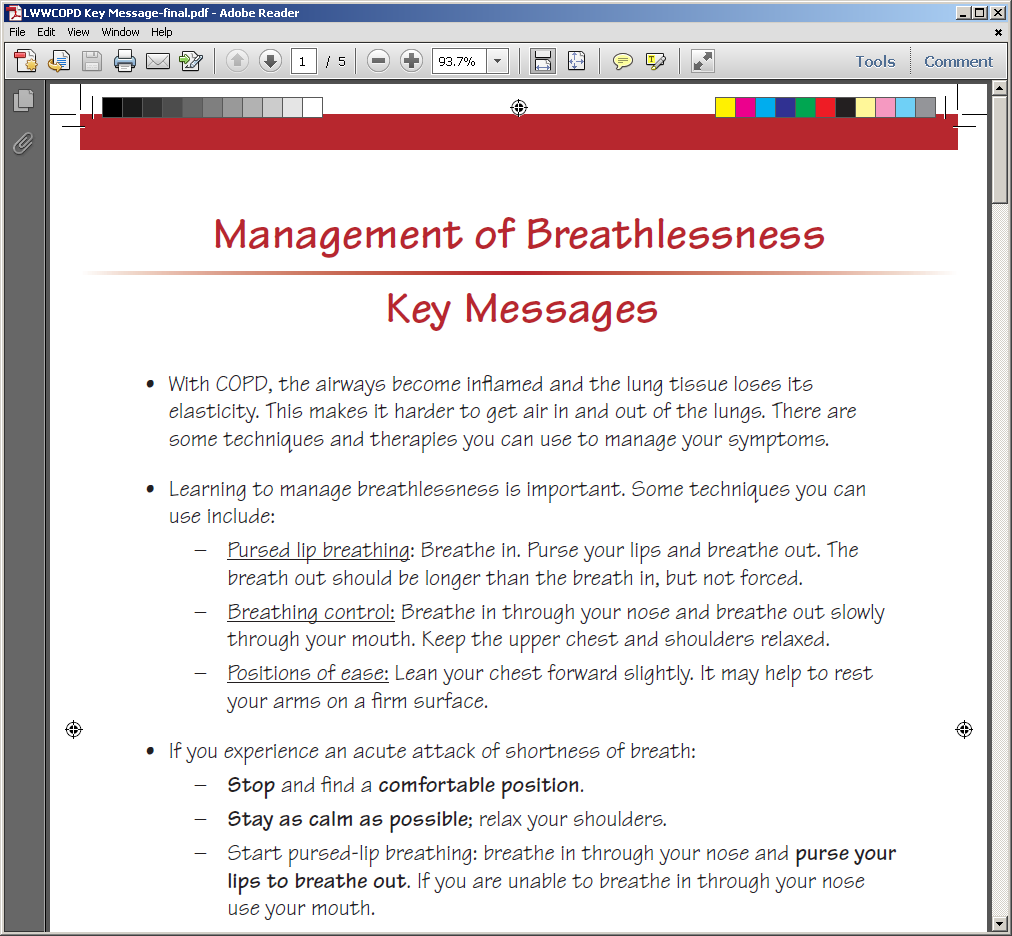 | **Key message sheets:** There are 5 key “take-home” messages and these are summarised at the end of the session – these are given to patients on an A4 sheet. |
| 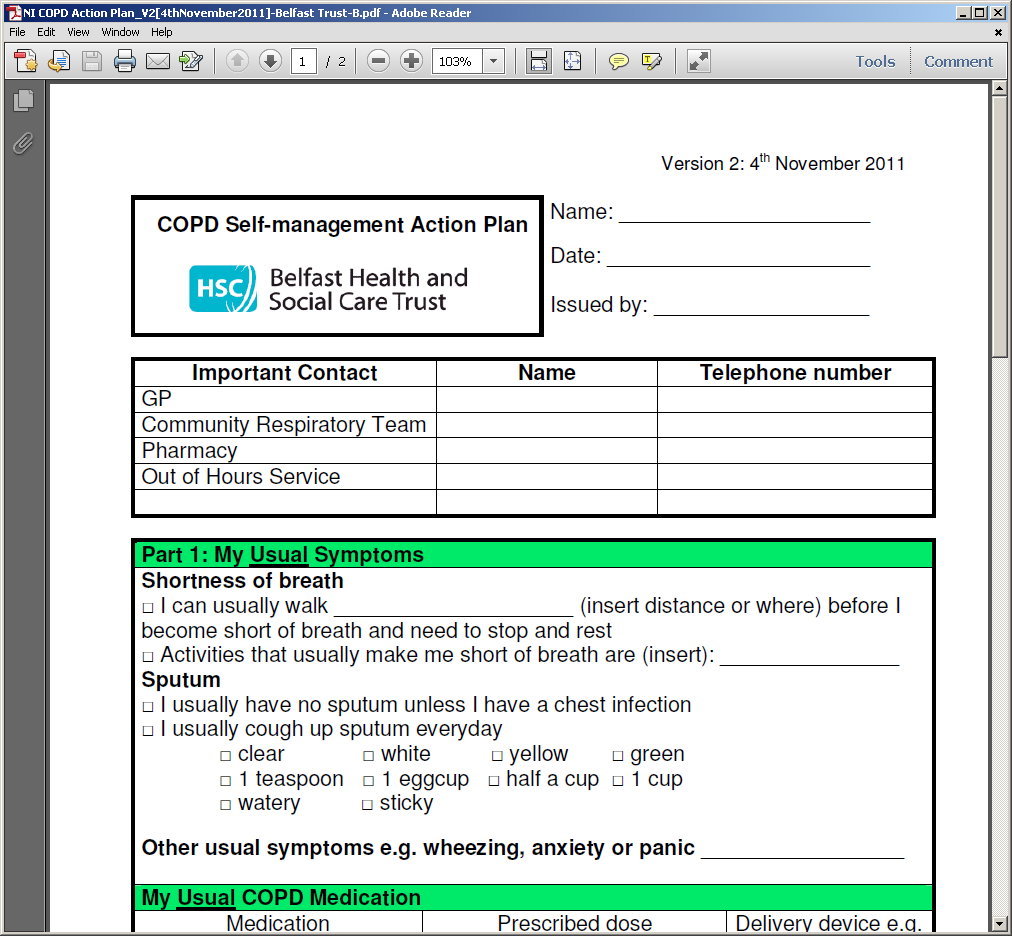 | **Action Plan:** The individualised Action Plan guides each patient in recognising and managing an exacerbation of their symptoms. |
