# Supplementary material for: Facilitating education in pulmonary rehabilitation using the Living Well with COPD programme for pulmonary rehabilitation: a process evaluation
Source: BMC Pulm Med. 2013 Aug 5;13:50. doi: 10.1186/1471-2466-13-50 (PMC3751129; doi:10.1186/1471-2466-13-50)
Supplement: Additional file 2: Table S2 — Elements of the process evaluation model assessed. [file 1471-2466-13-50-S2.doc]

**Additional file 2: Table S2:** Elements of the process evaluation model assessed

| **Process evaluation elements (and definition)** | **Data collection instrument** | **Completed by whom** | **Completion timescale** | **Assessments** |
| --- | --- | --- | --- | --- |
| Reach (proportion of the intended audience that participates in the intervention, and their attendance rates) | Attendance log | Health professional | Weekly after each education session | - Attendance at education sessions |
| Dose delivered (amount of the intervention delivered by facilitators) | Evaluation questionnaire | Health professional | Weekly after each education session | - Duration of the education session - Percentage of the session covered |
|  | Checklist | Health professional | Weekly after each education session | - Compliance with the cue cards - Compliance with the key messages / action plan |
| Dose received (extent to which participants actively engage with, interact with and/or use materials/resources) | Evaluation questionnaire | Health professional | Weekly after each education session | - Overall opinion of the session (five-point likert range: excellent to poor) - Satisfaction with the amount of practical information in the session (five-point likert range: very satisfied to very unsatisfied) - Satisfaction with the materials provided to deliver the session (five-point likert range: very satisfied to very unsatisfied) - Advantages and disadvantages of the programme - Areas of the programme for improvement |
|  | Evaluation questionnaire | Patient | Weekly after each education session | - Overall opinion of the session (five-point likert range: excellent to poor) - Satisfaction with the amount of practical information in the session (five-point likert range: very satisfied to very unsatisfied) - Aspects of the session they most and least enjoyed - Areas of the programme for improvement |
|  | Understanding COPD questionnaire (Section B) | Patient | End of the programme | - Satisfaction with practical information - Satisfaction of content of written information - Satisfaction with content of education sessions - Approachability of the health professionals - Accessibility of location - Can you suggest additional topics that should be included? |
| Fidelity (extent to which the intervention was implemented as planned) | Bristol COPD Knowledge Questionnaire | Patient | Start and end of the programme | - Change in knowledge |
|  | Understanding COPD questionnaire (Section A) | Patient | Start and end of the programme | - Change in understanding and self-efficacy |
