# Supplementary material for: Facilitating education in pulmonary rehabilitation using the Living Well with COPD programme for pulmonary rehabilitation: a process evaluation
Source: BMC Pulm Med. 2013 Aug 5;13:50. doi: 10.1186/1471-2466-13-50 (PMC3751129; doi:10.1186/1471-2466-13-50)
Supplement: Additional file 3: Table S3 — Health professionals’ feedback on the LWWCOPD programme for pulmonary rehabilitation. [file 1471-2466-13-50-S3.doc]

**Additional file 3: Table S3: Health professionals’ feedback on the LWWCOPD programme for pulmonary rehabilitation**

| **Feedback** | |
| --- | --- |
| **Positive feedback** | **Negative feedback / Areas for improvement** |
| **Good resources for the health professional**  “Prompting for health professionals”  “Easy to follow and deliver” | **Repetitive**  “Repetitive at times” |
| **Comprehensive, evidence-based content**  “Ensured all aspects are covered in detail”  “Evidence based ” | **Long sessions**  “A lot of information in one session” |
| **Good patient friendly materials**  “Easy for patients to understand whilst providing sufficient information” | **Poor materials**  “Visual material not adequate” |
| **Variety of teaching methods**  “Good mix of interactive lecturing, demonstration and group discussion”  “It is not on power point therefore more informal” | **Too many medical terms**  “Some content of script contained too much medical terminology” |
| **Structured, distinct education sessions with defined curriculum**  “Linked to other sessions”  “Well structured”  “Planned talk enables direction of group” | **Not suitable for other conditions**  “Not all patients attending have COPD, pulmonary rehabilitation needs to address the individual diagnosis” |
| **Can be delivered by any health professional**  “Provided by any discipline in thorough manner” | **Very scripted**  “Not individualised enough. It was too rigid.”  “Did not draw on own expertise as just reading script” |
| **Easier to deliver for the second time**  “Was easier this time around and delivered in a more relaxed fashion. More aware of links and reinforcing previous session delivered.”  “Ease of delivery was increased due to increased familiarity” | **Requires preparation and practice**  “Need to be very familiar with content as going between sheets, flipchart and posters can get confusing” |
|  | **Order of education sessions**  “Need medication sessions before action plan” |
|  | **Group dynamics**  “Reluctance for individuals to talk about embarrassing symptoms in front of small group” |
|  | **Content**  “I expanded a little on muscle wasting in COPD and why exercise was important and how muscles become more efficient as they get fitter etc” |
