# Supplementary material for: Facilitating education in pulmonary rehabilitation using the Living Well with COPD programme for pulmonary rehabilitation: a process evaluation
Source: BMC Pulm Med. 2013 Aug 5;13:50. doi: 10.1186/1471-2466-13-50 (PMC3751129; doi:10.1186/1471-2466-13-50)
Supplement: Additional file 4: Table S4 — Patients’ feedback during the evaluation of the LWWCOPD programme for pulmonary rehabilitation. [file 1471-2466-13-50-S4.doc]

Additional file 4: Table S4: Patients’ feedback during the evaluation of the LWWCOPD programme for pulmonary rehabilitation

| **Component** | **Feedback** |
| --- | --- |
| Improved knowledge and self-efficacy | **Managing COPD:** “I have a better understanding of COPD”, “It does help with coping with COPD” |
|  | **Managing breathlessness:** “Have more understanding of what to do when I have shorting of breath and panic attacks”, “Classes were first class in every aspect it helped to build up more confidence and breathe more calmly. It helped me to remain calm”, “I can walk further now knowing I will get short of breath but can control it” |
|  | **Conserving energy:** “Shows you how to breathe when lifting…… when walking……when going upstairs”, “Learning to know your capabilities” |
|  | **Managing exacerbations:** “To know the changes in your symptoms”, “Better understanding of action to be taken” |
|  | **Taking medications:** “Better understanding of how medications work”, “This has given me more confidence to get on” |
|  | **Managing psychosocial issues:** “That I can manage anxiety with help”, “That being depressed was part of my illness”, “Knowing what other groups are out there” |
|  | **Taking part in exercise:** “More ability to do exercise at own pace”, “The confidence to carry on with certain exercises” |
| Peer support | **Meeting other people with COPD:** “Meeting people with the same condition”, “Reassurance that I am not alone” |
|  | **Sharing of information:** “The exchange of information amongst the class”, “Finding out my problems are common” |
| Staff /  Atmosphere | **Friendly, approachable and helpful:** “Interaction between presenters and patients”, “I know I can approach staff for my needs” |
|  | **Fun/enjoyable:** “Good fun”, “Good atmosphere” |
| Content | **Clear, understandable and useful information:**  “All of the information was very useful”, “The talks are plain and not hard to understand (all in my words)” |
|  | **Interactive and practical demonstrations**: “Practical demonstrations on various techniques”, “SOS technique”, “The six principles of energy conservation will enable me to cope much better with daily activities”, “Finding how well relaxation my way works” |
|  | **Visual reinforcement:**  “Liked poster”, “Individual plans” |
| Suggestions for improvement | **Delivery:** “Perhaps the presentation should be screened”, “Perhaps requires a bit more preparation” |
|  | **Content:** “More relaxation techniques would be good”, “Use of inhalers in shortness of breath attack” |
|  | **Location:** “Very far to walk” |
|  | **Length of sessions:** “Perhaps the presentation was a bit long” |
|  | **Facilitators:** “If a doctor could come to the class” |
|  | **Supplementary materials:** “A leaflet to explain to partner more about COPD” |
|  | **Inclusion of family:** “Involvement of patients next of kin” |
|  | **Additional sessions:** “Importance of diet or maintaining weight” |
